# Diagnosis and prognosis of myocardial infarction in a patient without obstructive coronary artery disease during bronchoscopy: a case study and literature review

**DOI:** 10.1186/s12872-020-01458-5

**Published:** 2020-04-21

**Authors:** Menghuan Li, Yang Liu, Hui Wang

**Affiliations:** The First Affiliated Hospital of Nanjing Medical University, Guangzhou Road No.300, Nanjing, 210029 China

**Keywords:** Myocardial infarction, Myocardial infarction in the absence of obstructive coronary artery disease (MINOCA), Bronchoscopy, Case study

## Abstract

**Background:**

It is increasingly recognized that a significant proportion of patients suffered from acute myocardial infraction (AMI) who did not have obstructive coronary artery disease (≥50% diameter stenosis). The term “MINOCA (myocardial infarction with nonobstructive coronary arteries)” was coined for such entity, however, the exact mechanism of MINOCA is still unclear. Herein, we report a patient with MINOCA during bronchoscopy and further review the recent literature.

**Case presentation:**

A 65-year-old woman was hospitalized with the main complaint of chest tightness, nausea and vomiting for 30 min during bronchoscopy under local anesthesia. Immediate electrocardiogram (ECG) showed ST-segments elevation in leads V2–6 compared with those at admission, and the further evolvement of leads V2–3 into pathological Q wave. Serum cardiac biomarkers revealed high-sensitive cardiac troponin T (hs-cTnT) levels of 20.12 ng/L and 674.6 ng/L at the peak (normal range 0-14 ng/L). Emergency coronary angiography (CAG) showed only approximate 30% stenosis in the left anterior descending (LAD) ostium and 40% stenosis in the first diagonal branch (D1), with quantitative flow ratio (QFR) value for LAD of 0.96. Moreover, her echocardiographic examination presented new significant abnormal wall motion (anterior ventricular wall) with an estimated left ventricular ejection fraction (LVEF) of 62.1% after the cardiac attack. Thoracic enhanced CT scanning indicated no obvious sign of pulmonary embolism. Therefore, with confirmed AMI and the absence of significant coronary stenosis simultaneously, MINOCA was diagnosed with the prescription of dual-antiplatelet, statins, beta-blocker, angiotensin receptors antagonist, calcium channel blocker and nitrate. This patient had a good prognosis during a follow-up of 14 months.

**Conclusion:**

In this case, bronchoscopy might have caused extremely tense and anxious which led to a sympathetic hyperfunction and acute coronary thrombosis induced by plaque disruption and coronary artery spasm. QFR value is a feasible technique to evaluate the functional coronary stenosis and assist the diagnose of MINOCA. Also, the diagnosis of MINOCA deems an exploration of underlying causes for proper management and prognostic evaluation.

## Background

Acute myocardial infarction (AMI) is a life-threatening disease which requires urgent intervention. It has been proven that arteriosclerosis is a critical pathogenesis of AMI [1]. However, there are significant proportion of patients who will develop AMI without obstructive coronary artery disease (≥50% diameter stenosis). The term “MINOCA (myocardial infarction with nonobstructive coronary arteries)” was recently coined for such entity. It is characterized by changes of electrocardiography and elevation of cardiac biomarkers, as well as non-obvious angiographic coronary artery stenosis [2]. MINOCA is found in about 5–6% of all patients with AMI confirmed by coronary angiography (CAG). For the heterogenetic patients of MINCOA, although the underlying pathophysiological mechanisms are poorly understood, several possible mechanisms were proposed, including plaque disruption, coronary artery spasm, in-situ thrombosis, spontaneous coronary artery dissection, type 2 MI and microvascular dysfunction [3]. It is vital to exclude other possible causes for troponin elevation, such as Takotsubo cardiomyopathy, myocarditis, pulmonary embolism, etc. As a local anesthesia procedure, bronchoscopy could cause certain stress for patients, but the overall severe cardiovascular complications during bronchoscopy are rare [4]. There are limited cases of AMI during bronchoscopy in previous literature. In this report, we present a case of MINOCA during bronchoscopy.

## Case presentation

A 65-year-old woman who underwent right upper lung adenocarcinoma resection for 4 months and was found to have an elevated carcinoembryonic antigen (CEA) 2 days before admission. In order to determine whether the tumor had local recurrence, the bronchoscopy examination was scheduled. She had a history of hypertension for a year and did not take medication. No history of other chronic diseases such as diabetes, coronary artery disease (CAD) or stroke, and no history of cigarettes, alcohol or substance abuse were reported. Physical examination showed normal BMI of 22.73 kg/m^2^ without other significant findings.

**Fig. 1:**
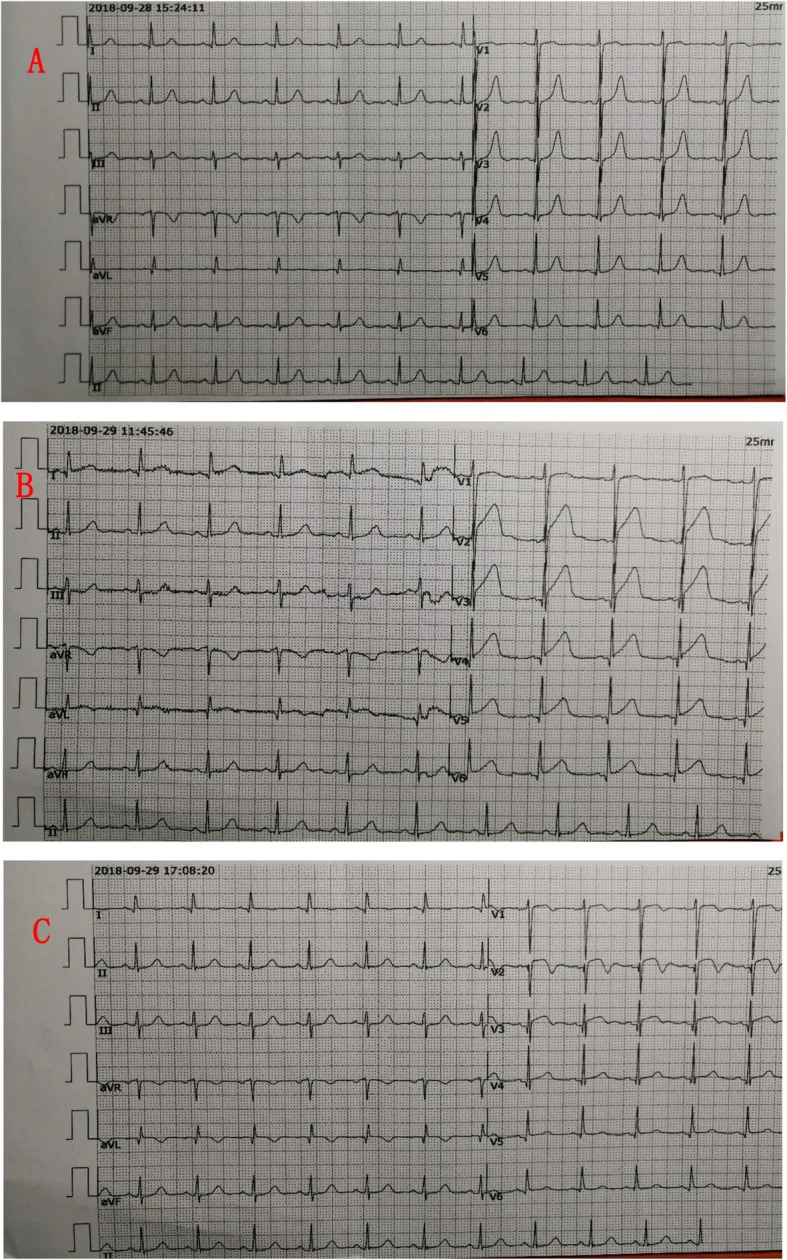
Dynamic changes in electrocardiography during the acute myocardial infarction. **a,** Sinus rhythm, normal ECG. **b**, ST-segments elevation in leads V2–6 during the sudden onset of cardiac attack. **c,** Pathological Q waves in leads V2–3, ST-segments elevation in leads V1–4, T-waves inverted in lead V1–3 and aVL

During the procedure, she had a sudden onset of chest tightness, nausea and vomiting for half an hour**.** An elevated blood pressure of 166/94 mmHg without other novel abnormal signs was found in the examination. Immediate electrocardiogram (ECG) showed ST-segments elevation in leads V2–6 compared with those at admission, then the further evolvement of leads V2–3 into pathological Q wave (Fig. 1). Laboratory result revealed high-sensitive cardiac troponin T (hs-cTnT) levels of 20.12 ng/L and 674.6 ng/L at the peak (normal range 0-14 ng/L). Moreover, her total cholesterol level was 6.70 mmol/L (normal range 3.00–5.70 mmol/L), low-density-lipoprotein cholesterol (LDL-C) level was 4.18 mmol/L (normal range 2.60–4.10 mmol/L), and lipoprotein (LP a) level was 1223 mg/L (normal range 0-300 mg/L). While other laboratory tests including routine complete blood count, urine test, glucose levels, renal and liver function, coagulation factors, hemoglobin A1c, thyroid function and autoimmune indicators were within the normal ranges. Thoracic enhanced CT scanning indicated no obvious sign of pulmonary embolism. Her echocardiographic examination presented new significant abnormal wall motion (anterior ventricular wall) with an estimated left ventricular ejection fraction (LVEF) of 62.1%**.**

**Fig. 2:**
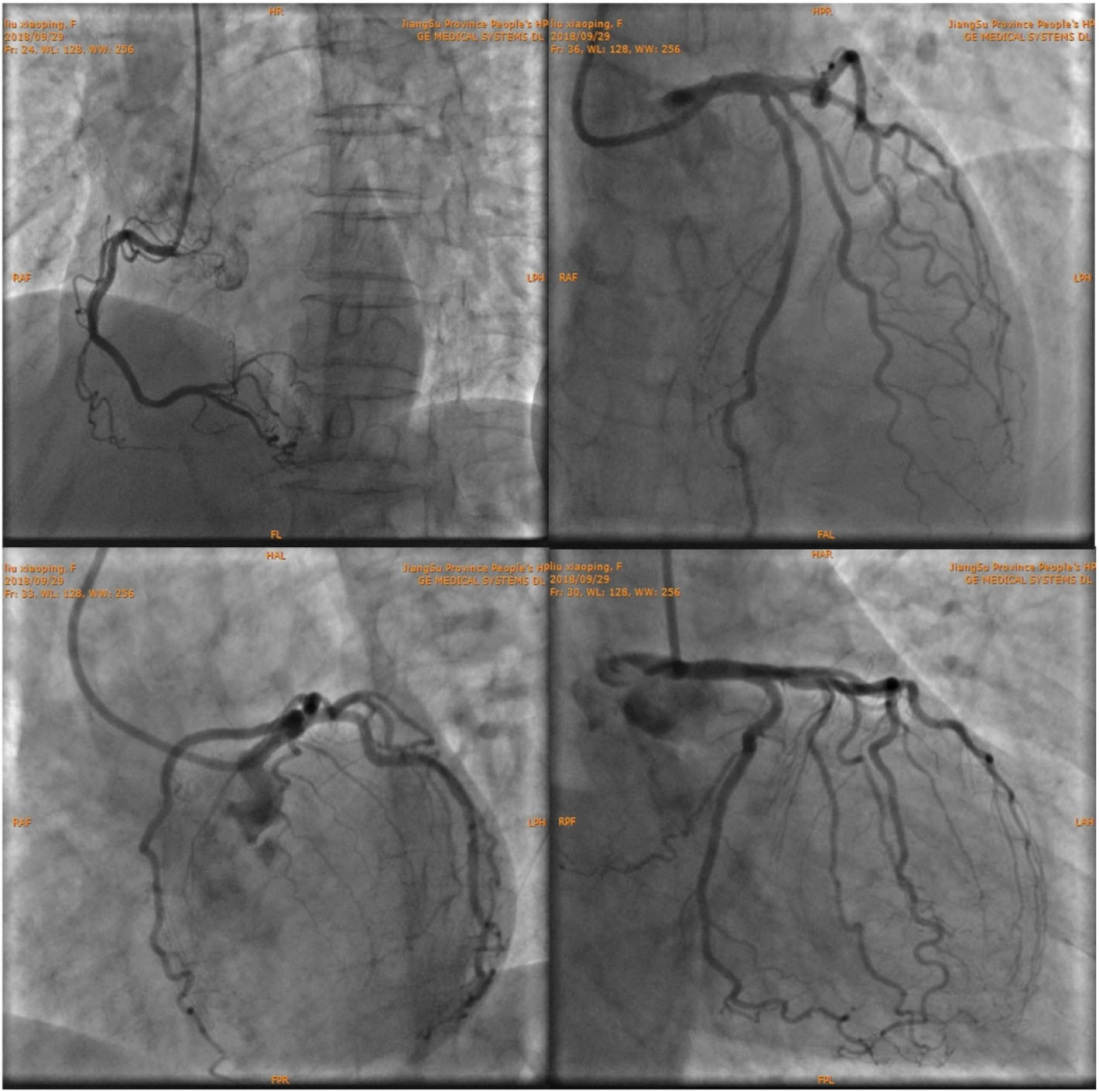
Images of coronary angiography. Approximate 30% stenosis in the left anterior descending (LAD) ostium and 40% stenosis in the first diagonal branch (D1)

**Fig. 3:**
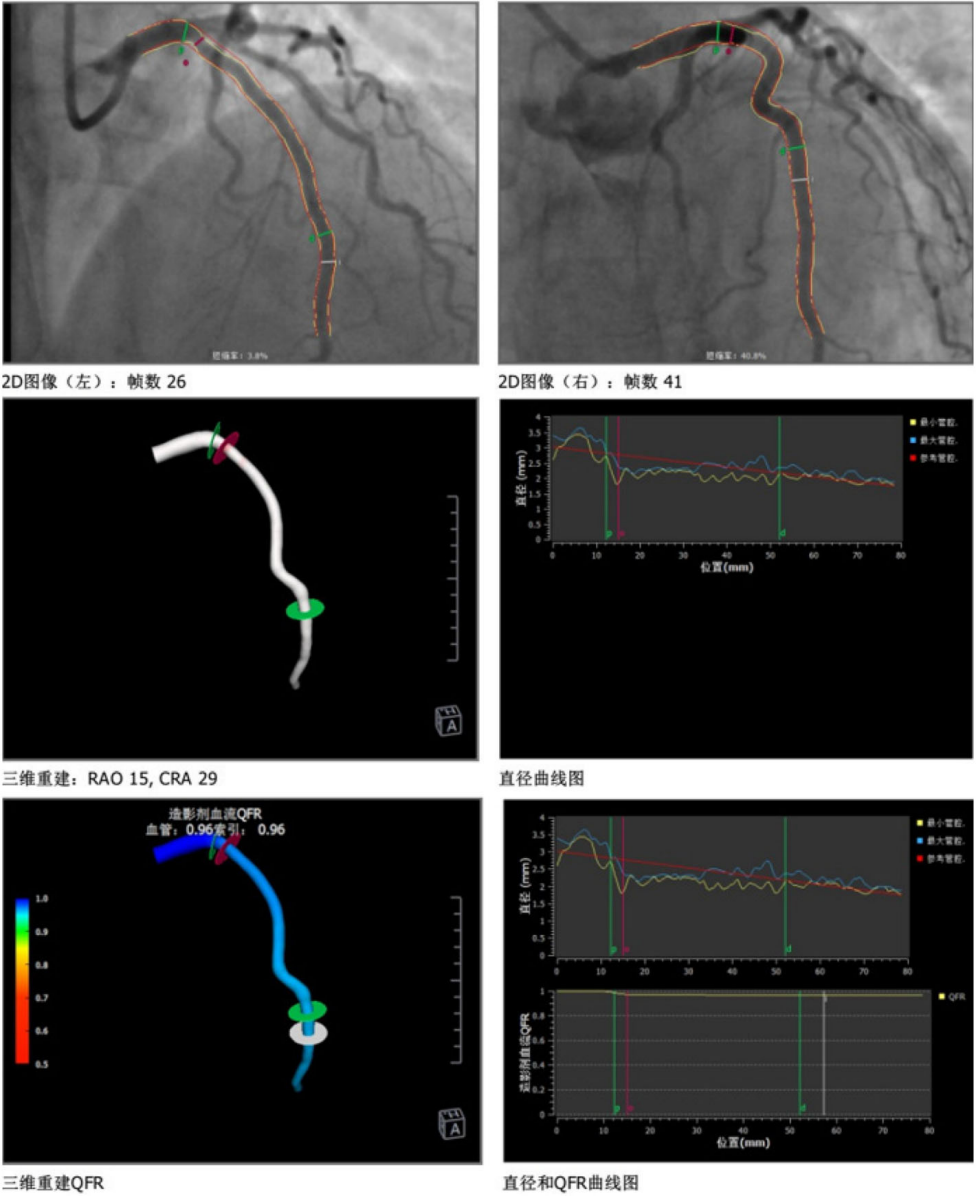
QFR value of left anterior descending (LAD). Based on the three-dimensional images from CAG and principles of fluid dynamics, QFR value of the target vessel - LAD was 0.96

The patient had atypical angina symptoms, dynamic changes of ECGs and elevated cardiac biomarker. Her echocardiographic showed new significant abnormalities in the wall motion. Emergency CAG was performed and showed only approximate 30% stenosis in the left anterior descending (LAD) ostium and 40% stenosis in the first diagonal branch (Fig. 2), functional quantitative flow ratio (QFR) value for LAD was 0.96 (Fig. 3). Combined with the above history and test results, the diagnosis of MINOCA was ultimately made with the suspected causes of stress-induced coronary artery spasm and plaque disruption.

She received dual anti-platelet therapy with daily low-dose aspirin for life and clopidogrel for 1 year. Considering her hyperlipidemia, statin and cholesterol absorption inhibitors were given. In addition, calcium channel blocker (CCB) and nitrites were given to control coronary artery spasm. Angiotensin receptor antagonist (ARB) was also prescribed for her uncontrolled blood pressure.

During a regular outpatient follow up for 14 months, she did not suffer from angina pectoris again. In the latest visit, echocardiograph showed that a normal ventricular wall motion and the estimated LVEF had rose to 69.4%. No major adverse cardiac events (MACE) including re-infarction, heart failure, stroke and all causes of death occurred during the period of follow-up.

## Discussion and conclusions

MINOCA is prevalent in 5–6% of all the AMI cases, and ranges from 5 to 15% depending on different studies [3]. The demographic and clinical features of MINOCA patients are different from those of AMI with obstructive coronary artery disease (AMI-CAD). MINOCA patients have a lower age and a higher incidence in women [5]. The fourth universal definition of myocardial infarction [6] demonstrated that AMI should be diagnosed with acute myocardial injury and myocardial ischemia simultaneously. Acute myocardial injury refers to the troponin >99th percentile of the upper reference level with a rise or fall in the serial assessments. And myocardial ischemia refers to the following manifestations: symptoms of myocardial ischemia, new ECG changes of ischemia, new Q waves in ECG, imaging evidence of surviving myocardial loss and regional wall motion abnormalities, or coronary thrombosis found by CAG or autopsy.

MINOCA is a working diagnosis and other ischemic disease and nonischemic disease that can mimic AMI, including sepsis, myocarditis, pulmonary embolism, missed obstructive CAD and Takotsubo syndrome etc., should be excluded [3]. The patient did not have any evidence of infection, so sepsis was excluded. She denied the prodromal virus-infected symptoms, and ECG showed typical changes of ischemia, so myocarditis was out of consideration. Moreover, her Thoracic enhanced CT scanning indicated no obvious signs of pulmonary embolism. Imaging of her CAG was confirmed by two different cardiologists who ensured no missed obstructive lesions.

Bronchoscopy is a routine diagnostic and therapeutic technique which is wildly applied for various pulmonary diseases for decades. The overall incidence of severe complications is low [4, 7] and it is considered a safe procedure with proper pre-evaluation. The most common complications during bronchoscopy are local anesthesia-related (0.3–0.5%), hypoxemia (0.2–21%), arrhythmias (1–10%), bleeding following biopsy (0.12–7.5%), pneumothorax or pneumomediastinum (1–6%) and fever (0.9–2.5%) [8]. Serious cardiac attacks associated with bronchoscopy are rare.

There are a few reports of takotsubo cardiomyopathy induced by flexible bronchoscopy which may be attributed to over-released catecholamines due to the extreme stress [9–12]. The manifestation of Takotsubo syndrome could be similar to AMI, and it is characterized by transient left ventricular dysfunction and ballooning of the apical part of the left ventricular with reduced LVEF [13, 14]. This patient was an elder woman who had a history of lung cancer resection and underwent bronchoscopy for check-up. During the procedure, she had atypical symptoms of myocardial ischemia, dynamic elevated cardiac biomarkers, typical ST-T changes and Q waves in ECG. However, no abnormalities at the apical part and impaired function of left ventricular were observed on her echocardiography. According to the fourth universal definition of myocardial infarction, we made a definite diagnose of AMI. Subsequent CAG showed no significant obstructive stenosis, thus we confirmed a diagnosis of MINOCA.

Epicardial coronary vasospasm can occur either in response to drugs or toxin that result in hyper-reactive of vascular smooth muscles, or in the disorders of coronary vasomotor tone [3]. These prolonged coronary vasospastic episodes can eventually lead to MINOCA. In one study, vasospasm occurred in 46% of the MINOCA patients who underwent provocative testing [15]**.** We believed that the patient might be in excessive stressed condition during the bronchoscopy, which trigger an excessive release of catecholamine causing severe and prolonged vasomotor dysfunction. The diagnosis of coronary vasospasm requires administration of intracoronary acetylcholine (Ach) and evaluation from invasive coronary angiography [16]. However, there are only limited experiences of Ach test in MINOCA patients. Furthermore, provocative spasm testing is seldom performed in China due to the scarce supply of Ach.

Coronary artery plaque disruption can trigger thrombus formation that results in AMI through distal embolization, or in some cases, transient complete in-situ thrombosis with spontaneous thrombolysis. Ouldzein et al. reported approximately 34% of plague rupture in a cohort of 68 patients with MINOCA [17]. The only definite diagnosis tool for plague disruption is intracoronary imaging, such as the higher-resolution optical coherence tomography (OCT) or intravascular ultrasound (IVUS). The underlying cause of this case was probably contributed to plague disruption, leading to acute thrombosis with spontaneous thrombolysis. Unfortunately, we have not been able to perform intracoronary imaging to figure out the possible mechanism, due to the cost and invasive method.

Fractional flow reserve (FFR) is a gold method to evaluate functional coronary stenosis, which was recommended in a scientific statement about MINOCA from American Heart Association (AHA) [3]. In recent years, quantitative flow ratio (QFR) has been validated as an accurate surrogate and showed an excellent diagnostic value for functional coronary stenosis compared with FFR [18–21]. Although our patient did not undergo provocative testing and intracoronary imaging for the assessment above, QFR was performed on the lesion of left anterior descending (LAD) as the alternative method which showed no significant functional stenosis of LAD supporting the diagnosis of MINOCA.

Previous studies had reported that dual anti-platelet agents had an incremental benefit to MINOCA patients with plaque disruption [22, 23]. Based on the results above, we prescribed her with aspirin and clopidogrel. Additionally, it was documented that CCB and short-acting nitrates were able to suppress the angina symptoms in vasospastic angina patients [16, 24], so CCB and nitrates were also prescribed. Angiotensin converting enzyme inhibitors (ACEI) /ARB and statin showed great cardioprotective effects for MINOCA patients [2], and thus both ARB and statin therapies were given as well. In one-year follow-up, the patient did not suffer from heart failure with a great recovery of ejection fraction.

Traditional risk factors for CAD are less prevalent in MINOCA patients, such as hypertension, hyperlipidemia, diabetes mellitus, smoking and drinking [25]. The patients of this case had two risk factors of hypertension and hyperlipidemia. The prognosis of MINOCA patients differed from those in many studies. Compared with AMI-CAD patients, most patients with MINOCA had a better prognosis [5, 26]. ACTION-Registry-GWTG study demonstrated that in-hospital mortality rate and MACE rate of MINOCA patients were lower than those of AMI-CAD patients [5]. However, VIGRO study indicated a similar 1-month and 1-year mortality rate between MONOCA and AMI-CAD patients [25]. ST segment elevation, heart failure or cardiac shock were independent predictors for in-hospital mortality rate [5]. However, it is worth mentioning that there was a lack of correlation between different etiologies in MINOCA patients.

The bronchoscopy might have caused extremely tense and anxious which led to a sympathetic hyperfunction and acute transient coronary thrombosis induced by plaque disruption and coronary artery spasm. The diagnosis of the MINOCA was considered according to the confirmed AMI and the absence of significant coronary stenosis simultaneously. And its diagnosis algorithm is so comprehensive that various diseases that mimic AMI should be excluded and underlying causes should be sought. QFR might be an efficient approach for the assessment of coronary artery. Overall, patients with MINOCA seems to have a better prognosis than those of AMI patients with obstructive coronary stenosis, which large cohort clinical trial is needed for further exploration of these heterogeneities.

## Data Availability

The data analyzed in the case report are not publicly available due to the privacy policy of the hospital but are available from the corresponding author on reasonable request.

## Abbreviations

MINOCA: Myocardial infarction with no obstructive coronary artery disease
ECG: Electrocardiogram
CAG: Coronary angiography
LAD: Left anterior descending
D1: The first diagonal branch
LVEF: Left ventricular ejection fraction
hs-cTnT: High-sensitive cardiac troponin T
ACEI: Angiotensin converting enzyme inhibitors
ARB: Angiotensin receptors antagonist
CCB: Calcium channel blocker
AMI: Acute myocardial infarction
CEA: Carcinoembryonic antigen
MACE: Major adverse cardiac events
CAD: Coronary artery disease
QFR: Quantitative flow ratio
CT: Computed tomography
LDL-C: Low-density-lipoprotein cholesterol
LP a: Lipoprotein a
Ach: Acetylcholine
OCT: Optical coherence tomography
IVUS: Intravascular ultrasound
AHA: American Heart Association
FFR: Fractional flow reserve
